# Sanitarium Bells

**Published:** 1911-02

**Authors:** W. J. Harvey

**Affiliations:** Denison, Texas


For Texas Medical Journal.

### Sanitarium Bells.

BY MRS. W. J. HARVEY, DENISON, TEXAS.

(PARODY ON EDGAR ALLAN POE'S BELLS.)

Hear the patients with the bells,—silver bells;
What a world of suffering their melody foretells!
How they tinkle, tinkle, tinkle,
In the icy air of night!
While the stars that oversprinkle
All the heavens, seem to twinkle
With the instrument and knife;
Keeping time, time, time,
In a sort of grusome rhyme,
To the tintinnabulation that so gloomily wells
From the bells, bells, bells, bells, bells,—
From the jingling and the tinkling of the bells.

Hear the convalescent's bells,—golden bells!
What a world of happiness their harmony foretells!
Through the balmy air of night

How they ring out their delight
From the molten-golden notes,
All in tune,
What a liquid ditty floats
To the turtle dove that listens, while she gloats
On the moon!
O, from out the sounding cells,
What a gush of euphony voluminously wells!
How it swells! how it dwells
On the future! how it tells
Of the rapture that impels
To the swinging and the ringing
Of the bells, bells, bells, bells, bells,—
To the rhyming and the chiming of the bells!

Hear the loud appendicitis bells,—brazen bells!
What a tale of terror, now, their turbulency tells!
In the startled ear of night
How they scream out their affright!
Too much anesthetised to speak,
They can only shriek, shriek,
Out of tune,
In a clamorous appealing to the mercy of the nurse,
In a mad expostulation with the deaf and frantic nurse
Screaming higher, higher, higher
With a desperate desire,
And a resolute endeavor,
Now—now to sit or never,
By the side of the pale-faced Mother.
O, the bells, bells, bells!
What a tale their terror tells
Of despair!
How they clang, and clash, and roar!
What a horror they outpour
On the bosom of the palpitating air!.
Yet the nurse, she fully knows,
By the twanging, the clanging,
How the danger ebbs and flows;
Yet the nurse distinctly tells,
In the jangling and the wrangling,
How the danger sinks and swells,
By the sinking or the swelling in the anger of the bells,—
Of the bells, bells, bells, bells, bells,—
In the clamour and the clangour of the bells!

Hear the tolling of the bells,—iron bells!
What a world of solemn thought their monody compels!
In the silence of the night,
How we shiver with affright
At the melancholy menace of their tone!
For every sound that floats
From the rust within their throats
Is a groan.
And the kin people, the kin people all,—
They that wait out in the hall,
All alone,
And who tolling, tolling, tolling,
In that muffled monotone,
Feel a glory in so rolling
On the nurse's heart a stone!
They are neither man nor woman,—
They are neither brute nor human,—
They are ghouls:
And their kin it is who tolls;
And he rolls, rolls, rolls, rolls
A paean from the bells!
And his heavy bosom swells
With the paean of the bells!
And he dances and he yells;
Keeping time, time, time,
In a sort of grusome rhyme,
To the tolling of the bells, bells, bells, bells, bells,—
To the moaning and the groaning of the bells.

